# Primary Angiitis of the Central Nervous System Presenting as a Cerebral Mass Lesion: A Case Report and Literature Review

**DOI:** 10.7759/cureus.8511

**Published:** 2020-06-08

**Authors:** Jacob E Bernstein, Stacey Podkovik, Samir Kashyap, Hammad Ghanchi, Ajay K Ananda

**Affiliations:** 1 Neurosurgery, Riverside University Health System Medical Center, Moreno Valley, USA; 2 Neurosurgery, Kaiser Permanente, Los Angeles, USA

**Keywords:** primary angiitis of the central nervous system, brain mass, craniotomy, vasculitis

## Abstract

Primary angiitis of the central nervous system (PACNS) is a rare form of vasculitis and is confined entirely to the central nervous system (CNS)without systemic involvement. We report a rare case of PACNS in a 39-year-old female with new onset seizures and a right frontal enhancing mass. Initially the patient was thought to have a high-grade glioma and thus underwent a right frontal craniotomy for resection of right frontal mass. Intraoperatively, two fresh tissue samples were sent for intraoperative consultation. Sample 1 showed predominantly necrotic tissue and scant glial cells while sample 2 revealed glial tissue favoring gliosis versus low-grade neoplasm with necrosis and a few acute inflammatory cells. Final pathological diagnosis was consistent with PACNS. Postoperatively, the patient recovered well from surgery with no neurological deficits and was discharged on postoperative day 3. Two weeks after surgery the patient was started on cyclophosphamide and prednisone by Rheumatology. At one month follow up, the patient remained asymptomatic and seizure free.

## Introduction

Primary angiitis of the central nervous system (PACNS) is a rare form of vasculitis with an annual incidence of 2.4 cases per 1,000,000 and is confined entirely to the central nervous system (CNS) without systemic involvement [1-10]. PACNS results in multifocal inflammation of the arteries and veins that may lead to ischemic or hemorrhagic infarcts in multiple vascular territories [1, 3-4]. Abnormalities are commonly seen in subcortical white matter, but may also involve deep gray matter, deep white matter, cerebral cortex, and leptomeninges [1, 3-4]. Headaches and encephalopathy are the most common presentation; however, patients can present with focal or systemic neurological deficits or seizures [1-2]. Subarachnoid hemorrhage and intracerebral hemorrhage occur in 10% of cases [1]. Cerebral mass lesions are a rare presentation that has been found to occur in 5%-15% of cases [1-2]. Here, we report a case of a woman who presented with new onset seizures and was found to have a right frontal mass concerning for high-grade glioma but was pathologically identified to be PACNS.

## Case presentation

**Figure 1:**
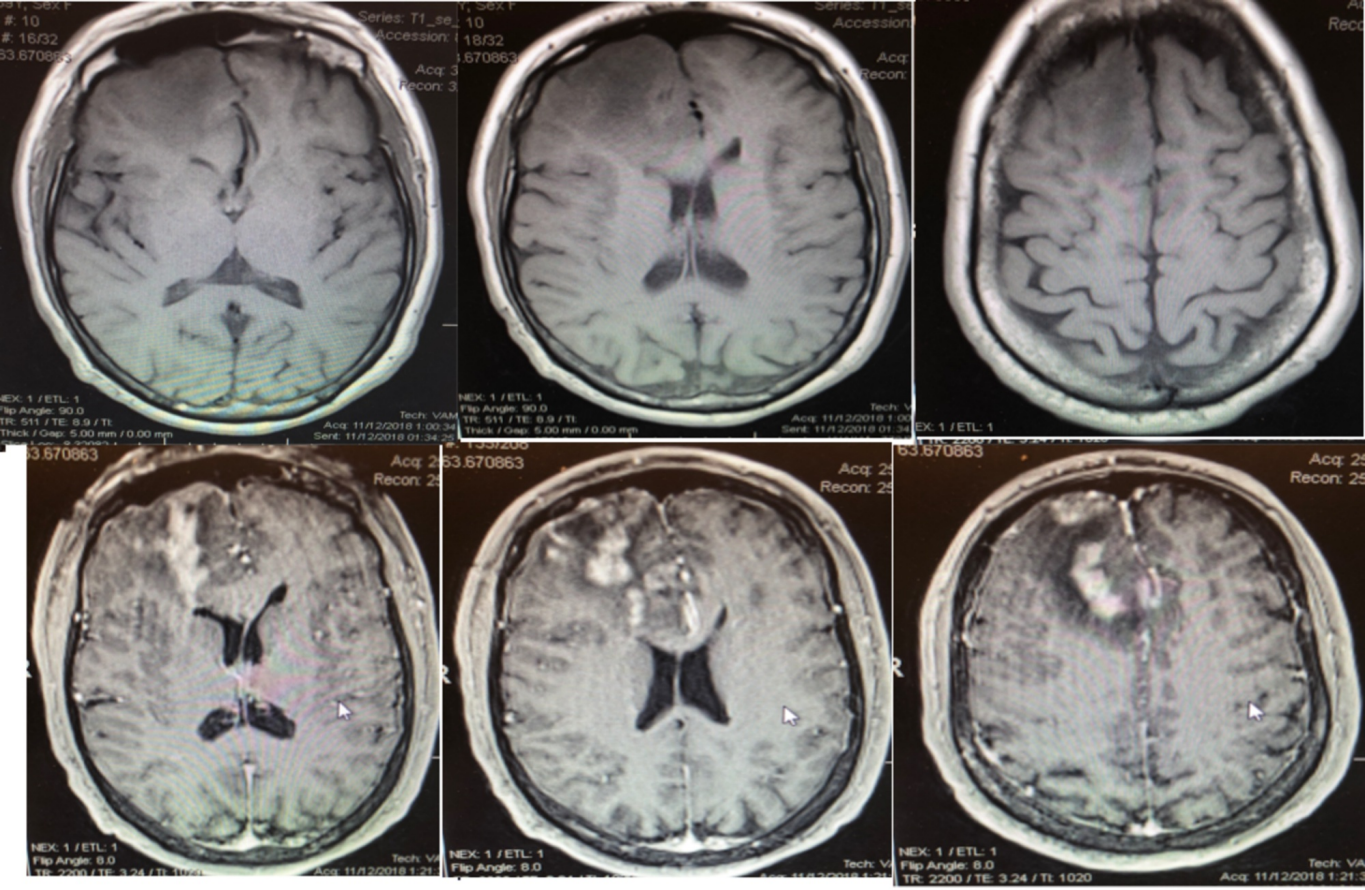
MRI with and without contrast showing large right frontal heterogeneously enhancing mass with surrounding vasogenic edema and mass affect on right lateral ventricle. Top Row: T1 axial without contrast Bottom Row: Axial T1 with contrast

**Figure 2:**
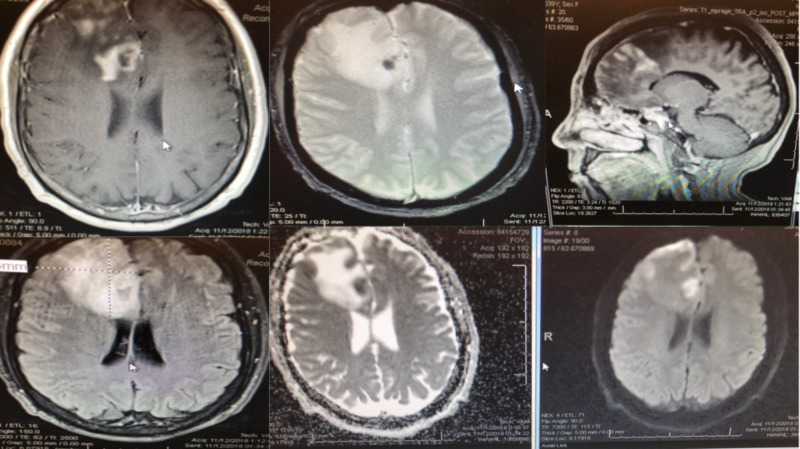
Preoperative MRI brain showing small areas of hemorrhage and diffusion restriction in medial frontal lobe. Top Left: Axial T2, Bottom Left: Axial fluid attenuated inversion recovery (FLAIR), Top Middle: Axial gradient echo (GRE), Bottom Middle: Axial apparent diffusion coefficient (ADC), Top Right: Sagittal T1 with contrast, Bottom Right: Axial diffusion weighted imaging (DWI)

**Figure 3:**
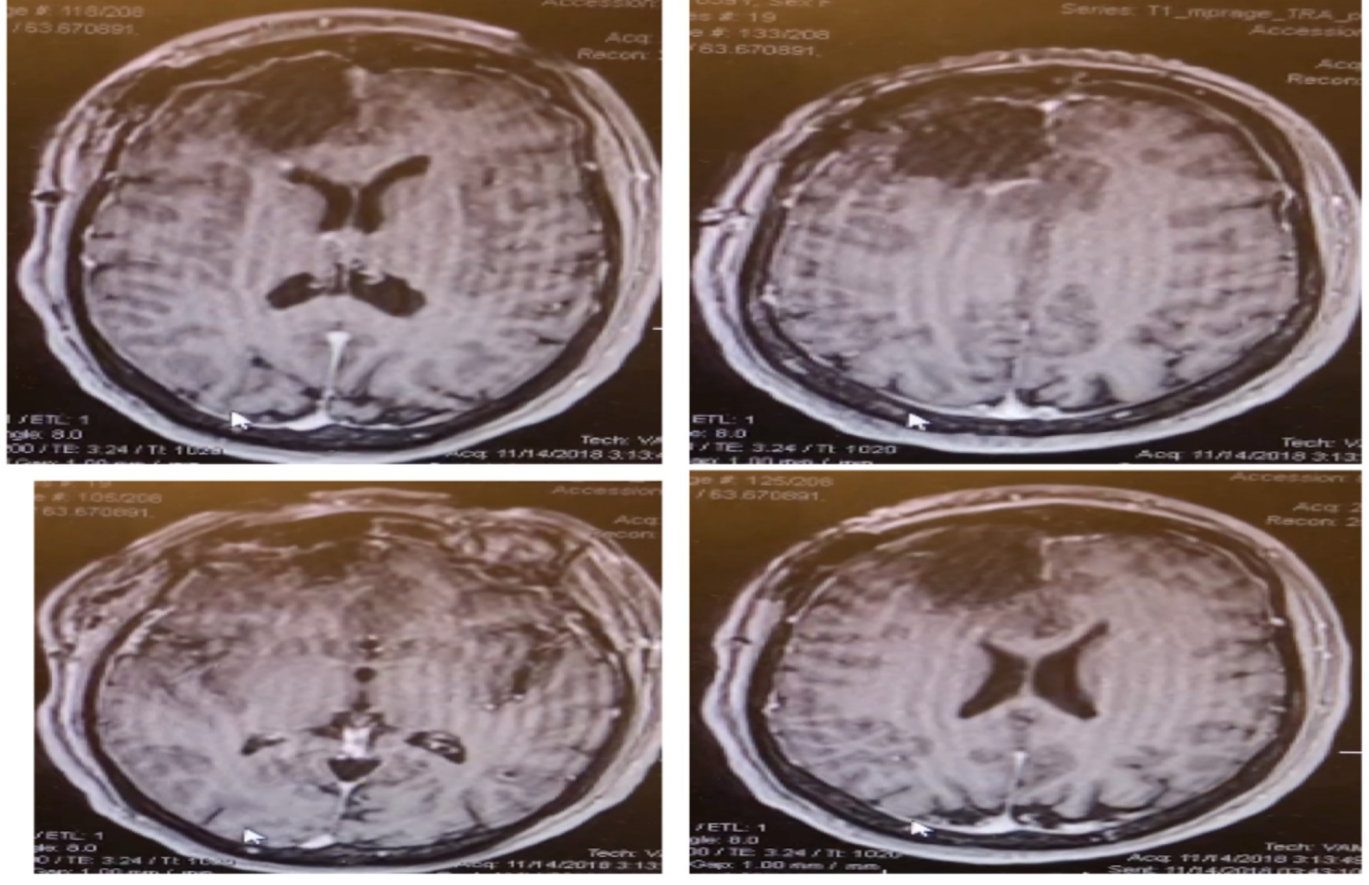
Postoperative MRI brain showing near total gross resection with tiny enhancement adjacent to right frontal horn. Images are T1 axial with contrast

The patient was a 39-year-old female with no significant past medical history who presented with a new onset of generalized tonic-clonic seizures. Her initial seizure resolved prior to arrival at the ED; however, the patient had a second seizure in ED that was aborted with two doses of lorazepam. The patient was loaded with 1 g of levetiracetam and started on 500 mg twice daily. The initial CT scan showed a large hypodense right frontal lesion without contrast enhancement (image not available). Once she recovered from her post-ictal period, she was alert and oriented to person, place, and time with no focal neurological deficits. The patient was started on dexamethasone 6 mg IV every six hours and admitted to ICU. MRI brain with and without contrast showed a large frontal heterogeneously enhancing mass with surrounding vasogenic edema, mass effect on right lateral ventricle, small amount of diffusion restriction in medial aspect of lesion, and small hemorrhage/calcification seen on gradient echo (GRE) (Figures 1-2). Her labs were unremarkable except for a C-reactive protein (CRP) of 12 (normal <8.0 mg/L) and erythrocyte sedimentation rate (ESR) of 8 (normal range 0-20 mm/h). The patient underwent a right frontal craniotomy for resection of mass on hospital day 2. Intraoperatively, two tissue samples were sent for intraoperative consultation. Sample 1 showed predominantly necrotic tissue and scant glial cells while sample 2 revealed glial tissue favoring gliosis versus low-grade neoplasm with necrosis and a few acute inflammatory cells. The patient’s postoperative neurological exam was unchanged as compared to the preoperative exam. MRI brain on postoperative day (POD) 1 revealed gross total resection (Figure 3). The patient had an uneventful recovery without seizures or neurological deficit and the patient was discharged home on POD 3.

 

Final pathological diagnosis

The lesion was predominantly angiocentric with transmural and perivascular lymphoplasmacytic infiltrates along with foci suggesting fibrinoid necrosis, and also associated infarcts. Immunohistochemistry results were positive for CD3, CD20, PAX 5, CD4, CD8, CD138, CD68, Ki67 and negative for kappa/lambda, EBV-EBER, HSV, CMV, SV-40. Final diagnosis was angiocentric lymphoplasmacytic infiltrates, consistent with PACNS. 

Follow up

Upon receiving pathology results, the patient was referred to Rheumatology for further treatment of PACNS. The patient was started on prednisone and cyclophosphamide two weeks after surgery. She was seen in neurosurgery clinic at two weeks and one month post-operatively, and was neurologically stable and without seizures.

## Discussion

We report a rare case of PACNS in a 39-year-old female with new onset of seizures and a right frontal mass. The patient was initially thought to have a high-grade glioma and underwent resection of the right frontal mass. Intraoperative diagnosis of the mass revealed necrosis and gliosis versus low-grade neoplasm prompting a full resection of the mass. However, the final pathological diagnosis was consistent with PACNS.

It typically occurs in the fourth and fifth decades with a mean age of onset of 50 years of age [1-4]. Mass-like lesions on brain imaging are a rare presentation and patients typically present with symptoms due to mass effect such as headache, nausea/vomiting, focal neurological deficit, and seizures [1-2]. Patients with mass-like lesions often have a shorter duration of symptoms prior to presentation than those without [2].

Diagnosis

PACNS is difficult to diagnose clinically or radiographically, often requiring a pathological diagnosis. It has a broad differential diagnosis as seen in Table 1. It most closely resembles reversible vasoconstriction syndrome (RVCS) [1, 10] (Table 2).

**Table 1:** Differential diagnosis for PACNS. PACNS, primary angiitis of the central nervous system; PRES, posterior reversible encephalopathy syndrome; ADEM, acute disseminated encephalomyelitis

Differential diagnosis of PACNS	
Mimic vasculitis	Reversible vasoconstriction syndrome, PRES, intracranial atherosclerosis, fibrous dysplasia
Arterial embolisms	Iatrogenic-catherization, cardiac shunt, endocarditis
Demylinating syndromes	ADEM, multiple sclerosis, sarcoidosis
Systemic vasculitis	Giant cell arteritis, polyarteritis nodosa, Churg Strauss, Wegener’s granulomatosis, lupus, sarcoidosis
Infection	Bacterial, tuberculosis, HIV, varicella zoster virus, herpes simplex virus, cytomegalovirus, fungal
Malignancy	Lymphoma, carcinomatosis meningitis, gliomatosis cerebri, myelodysplastic syndrome, paraneoplastic syndrome, glioma
Toxic	Amphetamines, cocaine
Thrombotic disorders	Antiphospholipid syndrome, disseminating intravascular coagulation, thrombotic micoangiopathy

**Table 2:** Comparison of PACNS and RVCS. PACNS, primary angiits of the central nervous system; RVCS, reversible vasoconstriction syndrome

PACNS	RVCS
Men more affected, mean age 40-60	Women more affected, mean age 20-140
Progressive	Acute onset
Transient ischemic attack (30%-50%), stroke, seizures, headaches (63%), encephalopathy	Severe thunderclap headache (mimicking headache of subarachnoid hemorrhage), transient ischemic attacks, stroke
No association with medications, migraines, drug use, or the peripartum period	Associated with syndromes or provocative agents associated with vasospasm such as medications, migraines, drug use, or peripuerum period
Variable improvement in angiographic findings	Angiographic vasospasm which improves over time
Increased cerebrospinal fluid white blood cell count and cerebrospinal fluid total protein	Normal cerebrospinal fluid
Treatment includes prednisone and cyclophosphamide	Treatment includes prednisone and calcium channel blockers

Diagnostic criteria for PACNS include the presence of an unexplained neurological deficit after clinical and laboratory evaluation, documentation by cerebral angiography, tissue examination of an arteritis within the CNS, and no evidence of systemic vasculitis or condition that would mimic its findings [9].

In patients with PACNS, MRI of the brain is abnormal in >90% of patients [1]. The most common MRI findings include T2/flair hyperintense subcortical white matter lesions but may also have abnormal signal changes in deep gray matter or cerebral cortex [1]. Ischemic infarcts are seen in about 50% of patients and commonly occur bilaterally in multiple vascular territories [1]. Subarachnoid hemorrhage and intracranial hemorrhage are seen in 10% of patients [1]. Mass lesions have been reported in up to 15% of cases [1]. Leptomeningeal enhancement has also been found in 10%-15% of cases [1]. The diagnosis cannot be made on MRI findings alone due to the variety of imaging characteristics seen in PACNS. Cerebral angiography has a sensitivity between 50% and 90% for diagnosis of PACNS and typically shows “beading” or multiple regions of stenosis in any given vessel with interposed regions of ectasia.

Cerebrospinal fluid studies are abnormal in 60%-90% of patients with PACNS and typically show mild to moderate elevations in total protein or white blood cell count which rarely exceeds 250 cells/uL [1-2]. Acute phase reactants such as ESR and CRP are found to be abnormal in 24% of cases. Biopsy is the gold standard for diagnosis [1-2, 5-6, 9]. However, due to the patchy distribution biopsies are only diagnostic in 50%-75% of cases [5]. Histological findings confirming diagnosis include transmural mononuclear inflammatory cell infiltration involving small vessels within brain parenchyma or leptomeninges with or without granulomas [5]. Granulomatous vasculitis is the most common histological pattern and may be associated with cerebral amyloid angiopathy [1-2, 5]. The granulomatous type includes both foreign body and Langerhans type giant cells with associated lymphocytes, plasma cells, and histiocytes [1]. Another common histological pattern is the lymphocyte pattern which is consistent with transmural and perivascular infiltrates causing fibrinoid necrosis and infarcts [1, 5]. Typical treatment of PACNS includes combination of corticosteroid and cyclophosphamide therapy [1-2].

The literature on surgical intervention for mass-like lesions of PACNS is limited due the rarity of the presentation, but limited results do show a trend towards increased benefit from resection [2]. Molloy et al. published a retrospective study showing 38 mass-like lesions out of 737 patients with PACNS (5%). Ten patients underwent surgical resection, of which five patients were in remission at median follow up of 24 months and three patients in remission at median follow up of nine months [2]. The two patients were noted to have died shortly after surgery without further explanation by the authors [2]. The results from Molloy et al. showed that surgical resection of PACNS mass-like lesions might be curative [2]. In our case, the patient presented with a new onset of seizures likely due to a right frontal enhancing mass that appeared to be resectable. The intraoperative pathological finding was not definitive, with low-grade neoplasm as a differential, a full resection was completed. The pathological diagnosis of PACNS was an unexpected finding in this case.

## Conclusions

We report a rare case of PACNS presenting as a cerebral mass lesion in a 39-year-old female with new onset of seizures and a right frontal mass. Surgical resection was associated with a good outcome in this patient. However, the patient still required prednisone and cyclophosphamide for definitive medical management.
